# Clinical and Imaging Features of Aortic Penetrating Atherosclerotic Ulcers: A Systematic Review and Meta-Analysis

**DOI:** 10.3390/jcm15031200

**Published:** 2026-02-03

**Authors:** Fatemeh Esfahanian, Mohammad Hossein Madani

**Affiliations:** 1 School of Medicine, Isfahan University of Medical Sciences, Isfahan 8174673461, Iran; fatemeh.esfahanian@gmail.com; 2 Department of Radiology, University of California, Davis Medical Center, 4860 Y St., Suite 3100, Sacramento, CA 95817, USA

**Keywords:** penetrating atherosclerotic ulcer, penetrating aortic ulcer

## Abstract

**Background/Objectives**: Penetrating atherosclerotic ulcer (PAU) is a type of acute aortic syndrome (AAS) characterized by an ulcer that penetrates from the inner lining into the middle layer of the aorta, often leading to serious complications such as intramural hematoma (IMH), aortic dissection, aneurysm, and rupture. PAU incidence has risen significantly in recent years. Advancements in imaging technologies like CT and MRI have improved early detection, yet the true prevalence remains unclear due to the asymptomatic nature of many cases. Thoracic endovascular aortic repair (TEVAR) is becoming the preferred treatment, but questions remain regarding its effectiveness in different clinical settings. This systematic review and meta-analysis aim to consolidate findings on PAU’s clinical presentation, imaging characteristics, and outcomes to improve diagnosis, risk assessment, and treatment strategies. **Methods**: PubMed, Scopus, Embase, and Web of Science (WOS) were systematically searched from 1994 until November 2023. Related data were collected and evaluated. We used a random-effect model to calculate a forest plot, a funnel plot, pooled prevalence, and publication bias by STATA 18. **Results**: Of 1179 studies, 56 met the inclusion criteria, and we analyzed 3023 PAU patients. The 30-day mortality rate was 4.4%, with a late mortality rate of 15.6%. According to our study, open surgery, pre-operation (pre-op) aortic rupture, post-operation (post-op) endoleak, distant year of publication, symptomatic patients, lesions in the ascending aorta, and greater diameter of the lesion were associated with mortality. TEVAR was the most common treatment (67.3%), the endoleak rate was 3.7%, and re-intervention occurred in 4.4% of cases. Significant heterogeneity and publication bias were noted across several outcomes. **Conclusions**: PAU primarily affects elderly males with cardiovascular comorbidities; interventions like TEVAR reduce short-term mortality; however, long-term outcomes remain challenging, which indicates further investigation is needed into early detection and treatment.

## 1. Introduction

Penetrating atherosclerotic ulcer (PAU) is a specific type of acute aortic syndrome (AAS). It is characterized by an ulcer-like projection that extends from the inner lining (intima) into the middle layer (media) of the aorta and often occurs at the site of a soft plaque. If left untreated, this condition can lead to serious complications such as intramural hematoma (IMH), aortic dissection, aortic aneurysm, pseudo-aneurysm, rupture, and potentially death [1,2].

Although PAU is less common than other forms of AAS, its reported incidence has increased markedly over recent decades, from approximately 0.6 to 2.6 per 100,000 person-years [3]. This rise is likely not reflective of a true epidemiologic surge but rather a consequence of advances in multimodality imaging methods such as transesophageal echocardiography, computed tomography (CT), magnetic resonance imaging (MRI), and aortography which have improved early detection, risk stratification, acute intervention planning, and potential complication evaluation [4,5]. These advancements may have led to the higher incidence rate of PAUs [3]. However, considering that most of these individuals are asymptomatic and that screening for PAU is not recommended, the true prevalence of PAU is largely unknown [6].

Management of PAU has evolved substantially with the increasing adoption of thoracic endovascular aortic repair (TEVAR), which has emerged as a preferred alternative to open surgery due to its minimally invasive nature, high success rates, reduced complication risks, and favourable long-term prognosis [6,7,8]. Despite these advantages, significant uncertainty remains regarding optimal patient selection, timing of intervention, and outcomes in elective versus emergency setting [9,10]. According to 2022 American Heart Association (AHA) guidelines for diagnosis and treatment of aortic diseases, the best choice for treating PAU mainly depends on associated complications [11].

Despite advancements in diagnostic imaging and treatment strategies, there are various clinical presentations and progressions of PAU, and determining patients who will benefit most from early intervention, especially among asymptomatic or high-risk individuals, remains a major clinical challenge. A comprehensive understanding of the clinical and imaging characteristics of PAU is crucial for improving diagnostic precision, guiding treatment strategies, and reducing associated morbidity and mortality.

This systematic review and meta-analysis aims to integrate previous studies regarding the clinical presentation, outcomes, and imaging characteristics of aortic PAUs. By collecting data from diverse studies, we seek to identify consistent patterns and clinically meaningful predictors that may support improved diagnostic accuracy, guide therapeutic decision-making, and ultimately enhance patient outcomes. Also, we present this article in accordance with the PRISMA reporting checklist.

## 2. Materials and Methods

The study protocol was registered with the Prospective Register of Systematic Reviews (PROSPERO registration number: CRD42024561130). This study is a meta-analysis and review of previous papers, not requiring permission from any particular entity. The Preferred Reporting Items for Systematic Reviews and Meta-Analysis (PRISMA) criteria were adhered to in this systematic review and meta-analysis [12].

### 2.1. Search Strategy

We searched PubMed, Embase, Web of Science, and Scopus systematically for relevant articles, with no date or language restrictions. We used the medical subject headings available in the National Library of Medicine related to penetrating atherosclerotic ulcers. Our detailed search strategy is available.

### 2.2. Study Selection

We screened titles and abstracts of relevant studies. This meta-analysis included studies on demographic characteristics, imaging parameters, outcomes, and mortality rates in patients with aortic PAUs. To avoid bias in analysis, case reports and case series with fewer than 10 patients were excluded. We excluded studies with insufficient data or written in non-English languages.

### 2.3. Data Extraction and Quality Assessment

Two reviewers extracted data using the Rayyan website for systematic review (Ouzzani Mouradand Hammady, 2016) [13].

Demographic data, symptoms, imaging features, treatment, anatomy, and prognosis were collected after removing duplicates.

The Newcastle–Ottawa scale (NOS) was used to assess the quality of non-randomized studies in this review [14], and studies with poor quality were excluded from the meta-analysis. Studies lacking standardized definitions for key outcomes were identified during quality assessment, and this limitation was reflected in lower NOS scores, potentially contributing to heterogeneity and affecting the interpretation of pooled results.

### 2.4. Statistical Analysis

We conducted a meta-analysis using STATA version 18 to estimate endoleak rates, re-intervention, 30-day mortality, and late mortality. We used a random-effect model and the single proportion command to calculate pooled estimates and 95% confidence intervals (CIs). Non-overlapping CIs indicated statistically significant differences. We first performed univariable meta-regression to explore the association between individual covariates and the primary outcome of 30-day mortality. Then, each covariate was tested independently to assess its effect on the mortality rate. Covariates with a *p*-value < 0.05 in univariable analysis were selected for inclusion in the multivariable meta-regression model. We evaluated heterogeneity using Higgins’ I2 statistic, with a value greater than 50% indicating significance. To assess publication bias, we used the Egger test and visually examined the funnel plot [15]. We used Duval and Tweedie’s Trim and Fill technique to adjust for publication bias and reconstructed the effect size. We considered a *p*-value of < 0.05 as significant.

## 3. Results

### 3.1. Literature Search Results

**Figure 1:**
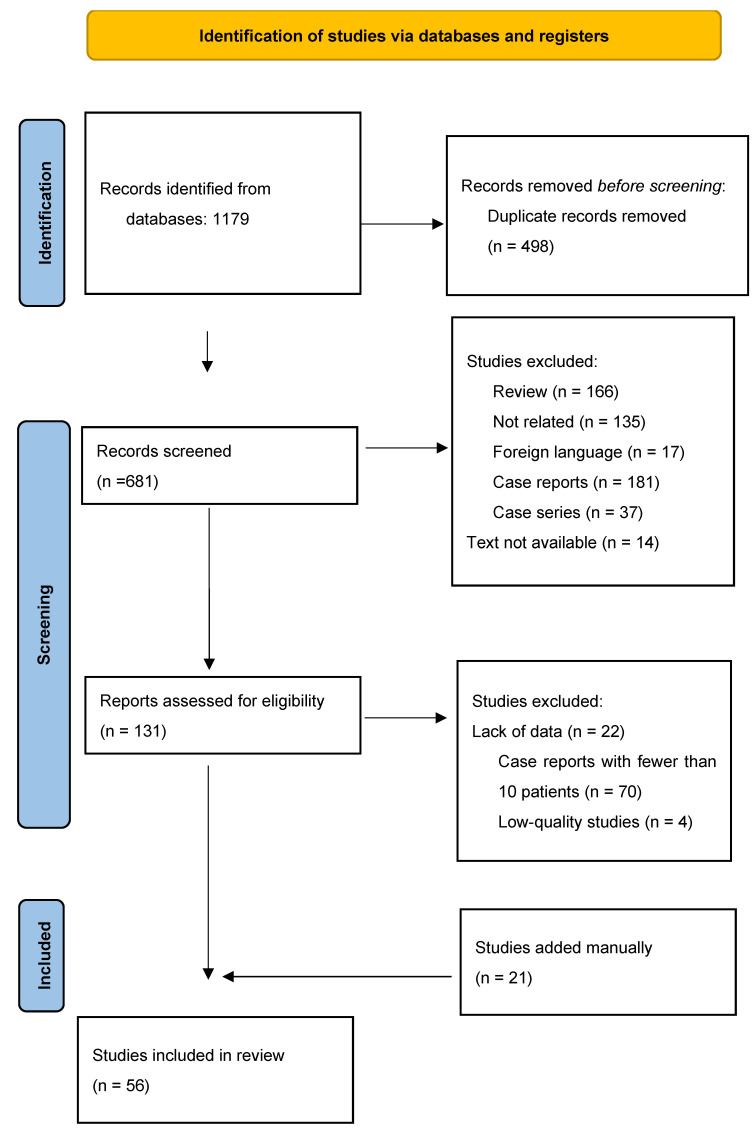
PRISMA flow diagram.

Of 1179 studies, 681 titles/abstracts were reviewed. We assessed 131 full texts for eligibility. Seventy studies reported less than 10 patients, twenty-two studies were excluded because of a lack of data on PAUs, and four had poor quality assessments. Finally, 56 articles with 3023 patients were included in the study. Data of included articles is available in the Supplementary Table S1. Figure 1 shows the Preferred Reporting Items for Systematic Reviews and Meta-analyses (PRISMA) flow diagram of study selection.

### 3.2. Meta-Analysis Results

Fifty-six studies were included in the meta-analysis, from 11 countries: 35.7% from the USA, 16.1% from Italy, 12.5% from Germany, 10.7% from China, and 7.1% from Austria, amongst others.

Data from 3023 patients who were diagnosed with PAUs were analyzed. The patient and lesion characteristics, co-morbidities, and outcomes are presented, and will be provided upon request.

Of the patients, 69.1% were male and 30.9% were female. Hypertension (HTN) was the most common comorbidity (83.6%), followed by hyperlipidemia (47.5%), smoking (43.9%), coronary artery disease (39%), chronic obstructive pulmonary disease (COPD) (23.3%), peripheral artery disease (18.2%), diabetes (15.6%), renal insufficiency (13.7%), and myocardial infarction (13.4%).

CT scan was the most frequent modality used for diagnosis (95.9%), followed by sonography (0.8%), echocardiography (0.6%), MRI (0.4%), and angiography (0.08%). The CT scan was ECG-gated in five percent of studies; four studies used 1.25 mm slices.

Different types of symptoms, such as back, chest, or abdominal pain, and shock, were presented in 36.8% of patients. Among associated complications, IMH was the most frequent one (18.5%), followed by thoracic aortic aneurysm (11.3%), distant abdominal aneurysm (9.9%), rupture (8.1%), dissection (1.7%), and thrombosis (0.6%). Regarding post-operative complication, in 5.3% of patients, endoleak occurred after procedures, followed by aneurysm and aortic dissection (Table 1).

PAU anatomical locations varied across different studies: descending thoracic aorta (DTA) (45.9%), abdominal aorta (39%), arch (15.1%), and ascending aorta (1.2%), respectively, were the most common sites. The percentages of anatomical zones of the aorta were not calculated because they were only defined in five studies. The greatest aortic diameter, lesion diameter, and depth are reported separately. Among imaging parameters, PAU lesion diameter was a significant risk factor for early mortality (*p* = 0.019).

The most frequent treatment reported in the literature was TEVAR (67.3%), followed by medical treatment for HTN (4.8%), conservative treatment (1.8%), open surgery (6%), and hybrid treatment (0.8%). Symptomatic patients (29.8%), size progression (16.7%), and rupture (12.8%) were reported as the most common indications among the 1629 patients who underwent TEVAR. Rupture (29.8%), size progression (3.4%), and symptomatic patients (4.1%) also reported in open-surgery cases. Among all patients, 13.4% of surgeries were performed in an acute setting.

### 3.3. Endoleak

**Figure 2:**
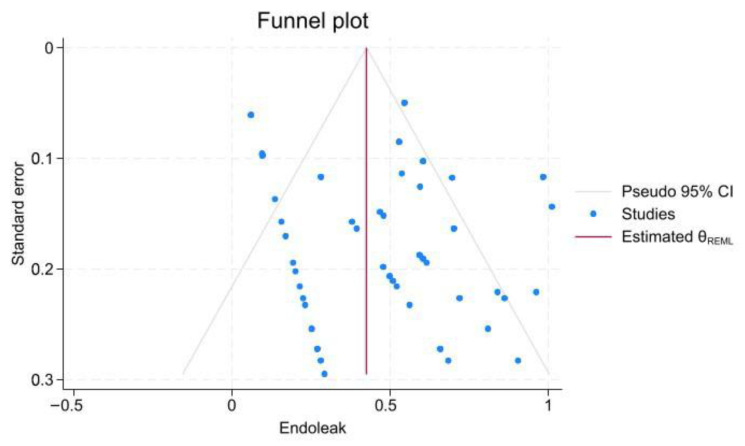
Funnel plot assessing publication bias in endoleak studies. This plot evaluates the symmetry of studies reporting endoleak incidence to identify potential publication bias.

Endoleak was recorded in 133 patients in 31 of 48 studies; however, in 4 studies, the type of endoleak was not reported. The most reported types of endoleak were type II (48.8%), type I (28.2%), type IV (8.3%), and type III (3%), respectively. The pooled prevalence was 3.7% (95% CI, 2.1–5.7; I^2^ = 70.33%). There was no statistically significant evidence of publication bias according to the Begg test (z = 0.67, *p* = 0.5; Figure 2 and Figure S1).

### 3.4. Re-Intervention

**Figure 3:**
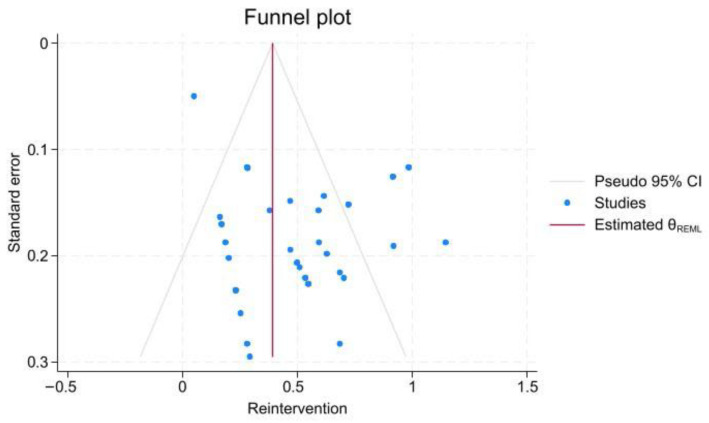
Funnel plot assessing publication bias in re-intervention outcomes.

In 53 studies, 1805 patients underwent intervention; however, during follow-up, 73 patients needed to undergo open or endovascular surgery following their primary intervention. Endoleak (45.2%) was the most prevalent cause of re-intervention, following by underlying atherosclerotic diseases (19%), malperfusion (5%), and disease progression (5%). The pooled prevalence was 4.4% (95% CI, 2.2–7.1; I^2^ = 68.64%) among 32 studies. The Begg test showed no evidence of publication bias (z = −0.36, *p* = 0.74; Figure 3 and Figure S2).

### 3.5. Mortality

**Figure 4:**
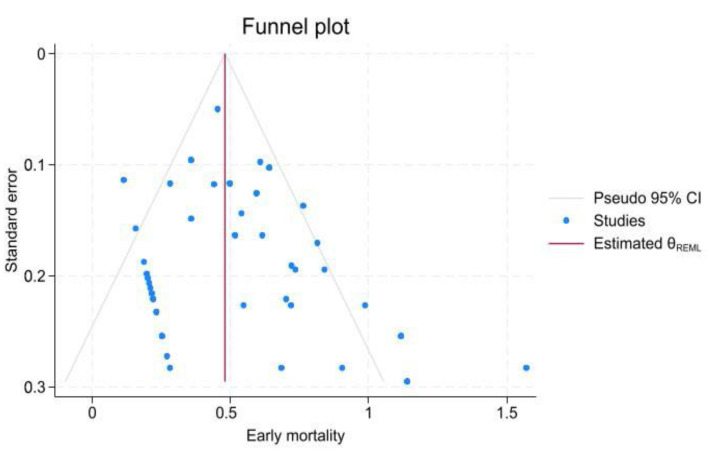
Funnel plot assessing publication bias for early mortality.

**Figure 5:**
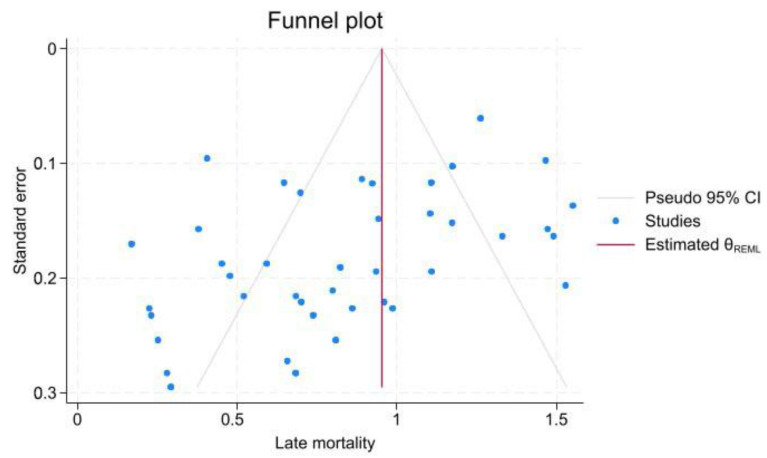
Funnel plot assessing publication bias for late mortality.

In the studies, a total of 515 patients died, with 110 deaths recorded in the 30-day follow-up period after hospitalization. The 30-day mortality rate was 4.4% (95% CI, 2.6–6.6; I^2^ = 62.69%) across 41 studies. Additionally, the mortality rate after 30 days (late mortality rate) was 15.6% (95% CI, 11.1–20.5; I^2^ = 83.87%) in 42 studies. According to 30-day mortality, there was a significant reduction in mortality among patients who underwent EVAR intervention vs. non-EVAR patients (3.51% vs. 13.60%, *p* < 0.001). The Beggs test revealed significant publication bias in the late mortality rate against early mortality (z = −2.38, *p* = 0.018; vs. z = 1.79, *p* = 0.073; Figure 4, Figure 5, Figures S3 and S4).

Meta-analysis of all four events showed significant heterogeneity (early mortality: Q(40) = 95.62, *p* value < 0.001; late mortality: Q(41) = 264.21, *p* value < 0.001; re-intervention: Q(31) = 141.92, *p* value < 0.001; endoleak: Q(47) = 176.23, *p* value < 0.001), indicating significant differences in effect sizes in this research. The I^2^ > 50% proved that the conflict is due to a different source than sampling error. We employed subgroup meta-analysis for inquiry into the source of variation. The data is available in Table 2.

The result of univariable meta-regression is available in Table 3. We used different covariates to build the most efficient model. The final model explained 76.46% of heterogeneity. In this model, a high rate of open surgery (*p*-value < 0.001), a high rate of pre-op aortic rupture (*p*-value = 0.001), a high rate of endoleak post-op (*p*-value < 005), and a more distant year of publication (*p*-value < 0.05) were associated with the 30-day mortality rate.

Publication bias was assessed using the meta-bias command, Egger method, and funnel plot. The Egger test showed significant publication bias in the 30-day mortality rate (*p*-value < 0.001), re-intervention rate (*p*-value < 0.001), and endoleak rate (*p*-value < 0.001), despite the late mortality rate (*p*-value = 0.48). Pooled prevalence estimates were computed, and Trim and Fill adjustments were performed for each kind of parameter to account for studies excluded because of publication bias. The results showed no significant changes.

## 4. Discussion

In this systematic review and meta-analysis, we review 56 studies from 1994 to January 2024 involving 3023 patients across 11 countries, revealing several key findings that enhance the understanding and management of PAUs. Our main finding is that PAU is a high-risk manifestation of atherosclerotic disease, with higher mortality in the long term than in the short term. It predominantly affects elderly men with risk factors such as hypertension. CT angiography is the main diagnostic tool used for early detection; while the increasing use of endovascular repair has substantially reduced early (30-day) mortality, our analysis demonstrates that long-term mortality remains considerable, indicating that PAU is a malignant condition requiring vigilant surveillance and long-term management rather than a problem resolved by acute intervention alone.

Atherosclerosis is the main cause of cardiovascular diseases (CVDs), which represent the main cause of mortality and morbidity worldwide, and PAUs are one of the presentations of CVDs. According to our findings, PAUs primarily impact men (69.1%), and a significant percentage of patients (83.6%) had hypertension, hyperlipidemia (47.5%), and were smokers (43.9%). Consistent with previous studies, hypertension, hyperlipidemia, and smoking are the major comorbidities in CVDs. Regarding gender disparities, differentiation could be related to the difference between major hormonal levels and genetic expression [16,17].

Various imaging modalities are available to diagnose acute aortic syndrome (AAS) such as MRI, CT, and echocardiography (TEE or TTE). The current gold standard in AAS imaging is CT angiography (CTA). CTA is widely available in most emergency departments, is non-invasive, requires fewer operators than ultrasound, and takes less time. The sensitivity of a CT scan in detecting AAS is over 95%, while the specificities range from 87% to 100% [18,19]. In our study, CT scans were the most commonly used diagnostic tool (95.9%), emphasizing their importance in identifying and evaluating PAUs. Despite CT being the most widely used imaging technique, the variability in imaging techniques (e.g., only 5% of studies using ECG gating; variations in slice thickness) suggests that standardization in imaging protocols could enhance diagnostic accuracy and consistency.

However, the prognosis for PAU patients remains debatable. As reported in our study, COPD and symptomatic PAU have a malignant clinical course, with a high risk of fatal complications [20,21]. Increased diameter of PAU, as reported by Pandey et al., is significantly associated with a higher mortality rate, which is consistent with our findings [22]. IMH is the most common complication at the time of diagnosis, as Cho et al. reported in 80% of patients [23], and may result in increased fragility of the vascular wall, but it was not associated with increased mortality [24]. Aortic rupture was the most fatal complication in our study, and according to Coady and Elefteriades, the prognosis for PAU rupture is worse than that of aortic dissection, with a 40% probability of rupture compared to 7% for type A dissection and 3.6% for type B dissection [25]. Similarly to previous findings, we demonstrated that pre- and post-operation aortic rupture is associated with a high 30-day mortality rate.

In the treatment of PAUs, various approaches such as open surgery, and medical, endovascular, and a combination of medical and surgical treatments have been used for many years. Among these different methods, according to Georgiadis et al., open surgery resulted in a mortality rate of up to 21% in treated patients [26]. During the acute phase, open surgery is a choice due to the instability of the aortic wall [27]; however, because of patients’ older age, underlying diseases such as liver and kidney dysfunction and coronary artery disease, cardiopulmonary insufficiency, and unstable condition, the mortality rate was significant [6,7,28].

Another concern in PAU is the long-term outcome. Previous studies reported various late mortality rates between 0.05 and 86% [29,30]. In our study, the calculated late mortality rate was 15.6%, significantly higher than the 30-day mortality. This indicates that despite the initial interventions, such as endovascular repair, which can provide short-term stabilization for the patient, long-term outcomes remain challenging. Regarding the heterogeneity in late mortality across studies (I^2^ = 83.87%), multiple factors, including PAU characteristics, symptomatic factors, risk factor modification, and complications, probably contribute to the progression of ulcers and this elevated rate [6,20,31].

Over the decades, the use of endovascular methods has expanded, and open surgery has been replaced by endovascular methods. As a result, endovascular repair has become the most commonly reported procedure, accounting for 67.3% of cases. Consistent with previous studies, symptom persistence, size progression, and rupture presence were the most reported indications for endovascular treatments. Also, our analysis showed that the increase in endovascular repair rate for the treatment of PAUs significantly decreases the 30-day mortality rate among patients because endovascular surgery generally requires only a femoral or iliac incision for exposure, short operative times, and no clamping of the aorta; however, the usage of endovascular repair could cause a complication named endoleak. This is the leakage of blood outside the stent graft. As we showed, endoleak is the most common complication (3.7%) and can increase mortality and cause re-intervention along with PAU progression. In our study about 4% of patients underwent endovascular re-intervention up to six years after the primary procedure due to ulcer progression and endoleak [9,32].

Our study demonstrated several implications for clinical practice. First, the substantial link between PAUs and classic atherosclerotic risk factors and male sex promotes focused risk stratification, especially for older patients with high blood pressure and smoking who have chest or back discomfort. Second, the widespread use of CT angiography in our research supports CTA as the primary diagnostic tool for suspected PAU. Our findings also show that standardized imaging techniques are needed to increase diagnostic consistency and discover problems early on. Third, the reduced short-term mortality associated with endovascular repair advocates for an endovascular-first treatment approach in anatomically appropriate patients, particularly those with elevated operative risk. Finally, the significantly elevated late mortality in contrast to 30-day mortality emphasizes that PAU is a chronic and potentially progressive illness. This illustrates the significance of continuous imaging monitoring, prompt detection of endoleak or ulcer advancement, and rigorous modification of cardiovascular risk factors as essential elements of treatment for patients. Based on the evidence we have, we suggest that patients have organized follow-up with CT angiography at 1 month, 6 months, and once a year after endovascular intervention. However, the schedule should be changed based on the patient’s risk factors and local protocols. This method makes it easier to find problems like endoleak, ulcer development, or the necessity for re-intervention early [33].

This systematic review and meta-analysis have various advantages. There are few studies that use meta-analysis to quantify prevalence estimates of endoleak, re-intervention, and mortality obtained from a comprehensive search technique. Despite previous studies, we did not limit our search to a particular location of PAU. However, this study has some limitations. First of all, some parameters such as past medical histories and aortic zone were not reported in all studies. This limits our analysis. Also, the majority of studies were retrospective. While the included studies cover multiple geographic regions, differences in healthcare infrastructure, imaging availability, and treatment practices may limit generalizability; nevertheless, the overall trends in risk factors, interventions, and outcomes are likely applicable across diverse settings with appropriate contextual adaptation. Although inconsistent reporting prevented stratified analysis, outcomes after acute interventions seemed poorer than those after elective procedures. This emphasizes the necessity of uniform reporting of intervention timing in future research and emphasizes the significance of early diagnosis and elective action where practical.

## 5. Conclusions

PAU is a common condition that primarily affects elderly males with hypertension, hyperlipidemia, and smoking. Endovascular intervention is the most frequent treatment and has been associated with a significant reduction in 30-day mortality rate. However, the higher late mortality rate indicates that PAU is a malignant and progressive condition despite initial intervention. These results highlight the importance of thorough patient selection, an initial endovascular approach for suitable patients, and obligatory long-term imaging surveillance in combination with strict cardiovascular risk-factor adjustment to enhance long-term outcomes. It should be noted that the evidence is limited by the predominance of retrospective studies, heterogeneity across studies, and variability in imaging and intervention protocols.

## Figures and Tables

**Table 1:** Post-operative complications after PAU intervention.

Complication	Pooled Prevalence (%)
Endoleak	5.3
Aneurysm formation	1.9
Aortic dissection	1.6
Aortic rupture	1.4
Stroke	1.2
Thrombosis	1.04
Myocardial infarction	0.6
Lymphatic fistula	0.04

**Table 2:** Subgroup analyses and between-study heterogeneity.

Outcome	Subgroup	No. of Studies (N)	Prevalence (95% CI)	I^2^ (%)	*p* (Heterogeneity)	*p* (Subgroup Difference)
Overall	—	40	0.044 (0.026–0.066)	62.69	—	—
Study type	Prospective	15	0.038 (0.009–0.080)	54.45	<0.001	0.215
	Retrospective	24	0.048 (0.006–0.075)	67.59	<0.001	
Era	<2010	18	0.078 (0.036–0.132)	64.65	<0.001	0.005
	2010–2019	11	0.047 (0.020–0.083)	52.79	0.015	
	≥2020	9	0.020 (0.008–0.037)	19.99	0.444	
Study quality	Good	34	0.044 (0.028–0.063)	47.73	0.001	0.650
	Fair	5	0.066 (0.000–0.228)	85.69	<0.001	
Intervention	EVAR	19	0.065 (0.027–0.114)	76.72	0.002	<0.001
	Open	26	0.028 (0.013–0.046)	26.94	0.062	
	Hybrid	33	0.038 (0.018–0.065)	68.60	<0.001	
	Non-surgical	31	0.033 (0.020–0.049)	25.56	0.056	
Gender	Male	55	0.685 (0.643–0.725)	78.46	<0.001	<0.001
	Female	55	0.315 (0.275–0.357)	78.46	<0.001	
WHO region	Europe (EUR)	22	0.034 (0.022–0.048)	0.00	0.130	0.392
	Americas (AMR)	14	0.065 (0.027–0.114)	68.91	<0.001	
	Western Pacific (WPR)	5	0.038 (0.000–0.198)	88.94	<0.001	

Abbreviation: EUR: Europe Region; AMR: Americas Region; WPR: Western Pacific Region.

**Table 3:** Univariate meta-regression analysis for mortality.

Variable	*p*-Value	R^2^ (%)	Wald χ^2^
Publication year	<0.001	35.7	19.36
Mean age	0.85	0.0	0.03
Male proportion	0.73	0.0	0.12
COPD	0.01	18.16	6.55
Smoking	0.27	0.0	1.21
Peripheral artery disease	0.31	11.14	1.03
Hyperlipidemia	0.66	0.0	0.18
Hypertension	0.57	0.21	0.32
Coronary artery disease	0.58	0.0	0.30
Diabetes mellitus	0.11	1.58	2.55
Myocardial infarction	0.39	0.0	0.72
Renal insufficiency	0.33	0.63	0.93
Symptomatic presentation	0.001	35.73	10.51
Lesion diameter	0.019	23.95	5.47
Lesion depth	0.053	20.03	3.74
Intramural hematoma	0.86	0.0	0.03
Rupture	<0.001	44.02	25.47
Endoleak	0.76	0.0	0.09
Open surgery	<0.001	50.13	30.42

Abbreviation: COPD, chronic obstructive pulmonary disease; IMH, intramural hematoma.

## Data Availability

The authors confirm that the data supporting the findings of this study are available within the article and its Supplementary Materials. Raw data that support the findings are available from the corresponding author upon reasonable request.

## Supplementary Materials

The following supporting information can be downloaded at: https://www.mdpi.com/article/10.3390/jcm15031200/s1, Figure S1. Forest plot of endoleak incidence across included studies. This figure presents a meta-analysis of endoleak rates following treatment. Each horizontal line represents a study’s confidence interval, and the diamond shows the pooled estimate; Figure S2. Forest plot of re-intervention rates across included studies. The figure illustrates the pooled incidence of re-intervention following treatment, with individual study estimates and overall summary effect; Figure S3. Forest plot of early mortality rates (≤30 days) across studies. This figure displays the early post-operative mortality rate from each study and the overall pooled estimate; Figure S4. Forest plot of late mortality rates (>30 days) across included studies; Table S1: Data of included articles; File S1: PRISMA_2020_checklist.
