# Supplementary material for: Clinical and Imaging Features of Aortic Penetrating Atherosclerotic Ulcers: A Systematic Review and Meta-Analysis
Source: J Clin Med. 2026 Feb 3;15(3):1200. doi: 10.3390/jcm15031200 (PMC12897750; doi:10.3390/jcm15031200)
Supplement: Supplementary file 1 [file jcm-15-01200-s001.zip › jcm-4071923-supplementary figures.pdf]

Journal of
Clinical Medicine

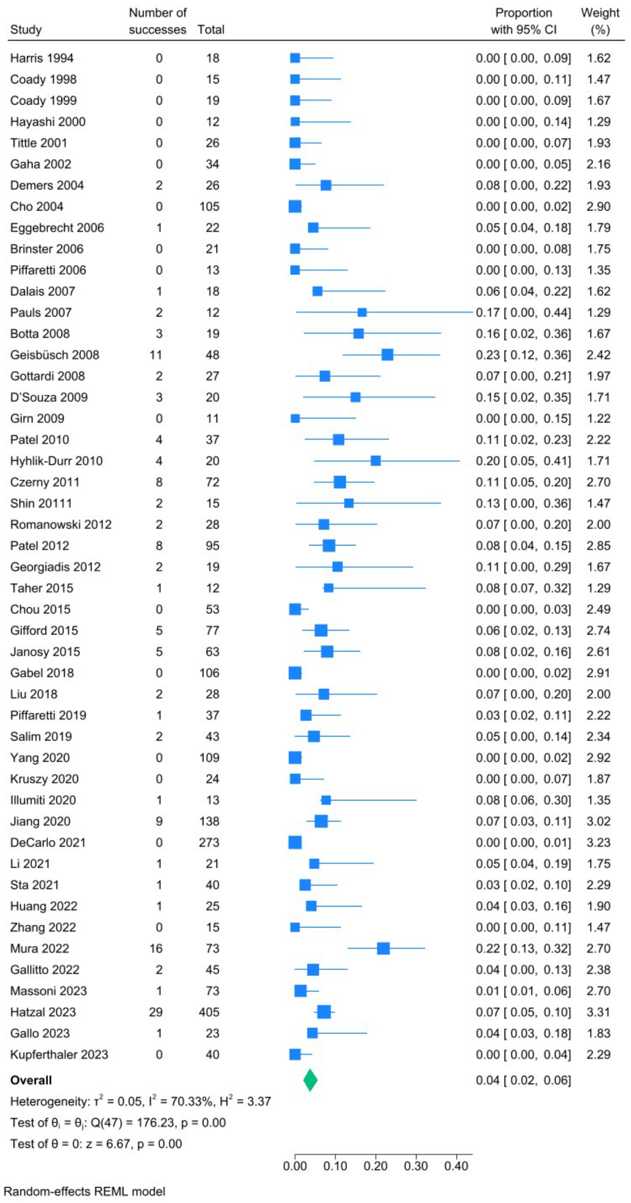

Figure S1. Forest plot of endoleak incidence across included studies [7–53]. This figure presents a meta-analysis of endoleak rates following treatment. Each horizontal line represents a study's confidence interval, and the diamond shows the pooled estimate.

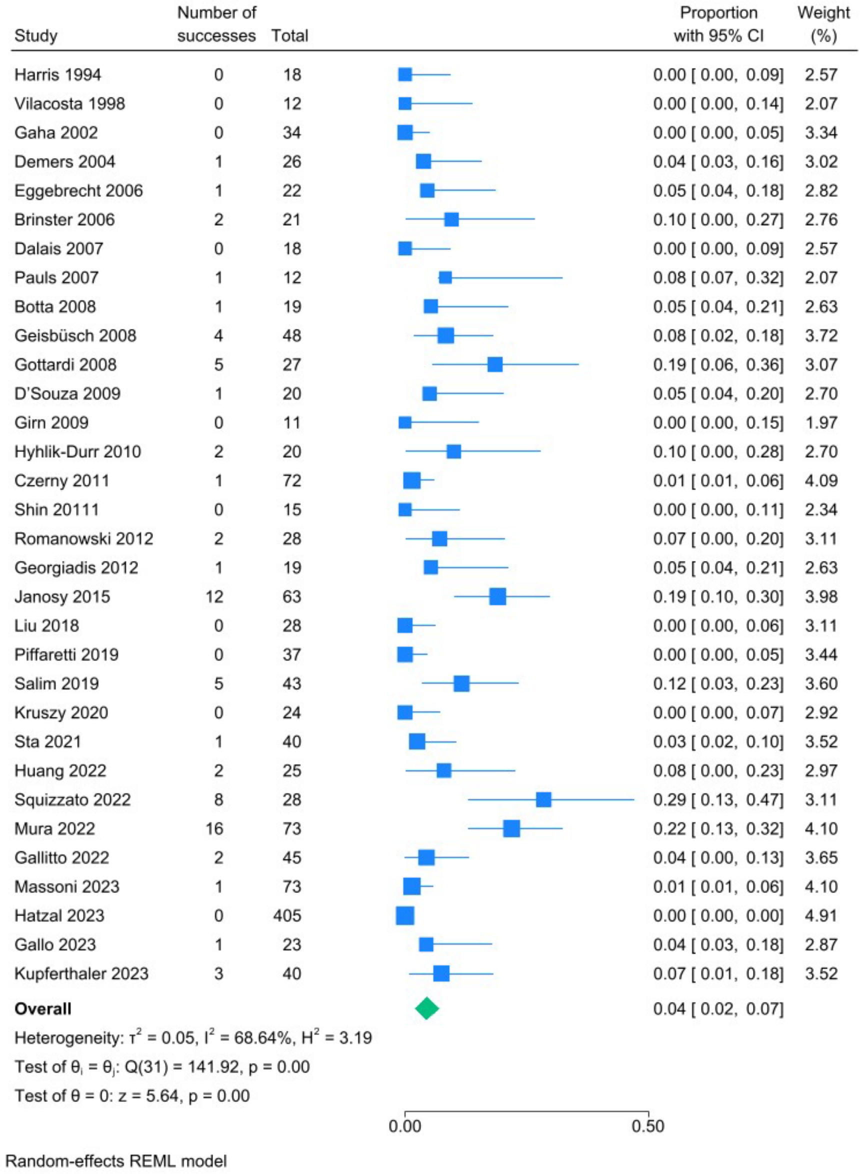

Figure S2. Forest plot of re-intervention rates across included studies [7,9–11,13–22,24,29,30,32,34,38–46,48,51,54,55]. The figure illustrates the pooled incidence of re-intervention following treatment, with individual study estimates and overall summary effect.

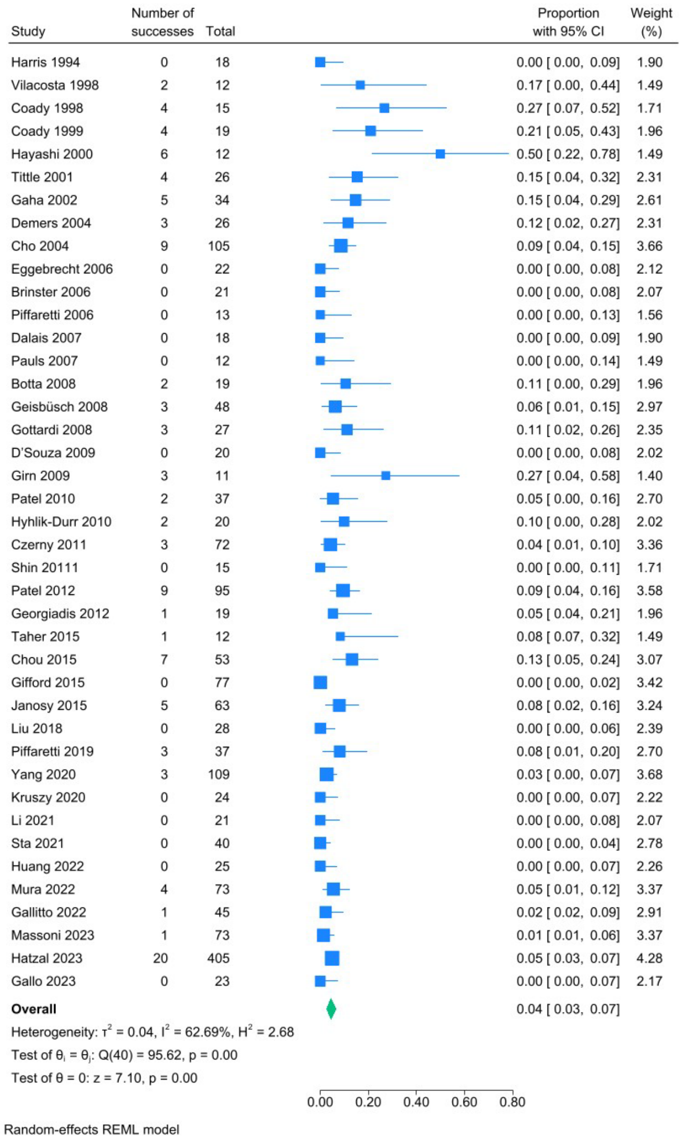

Figure S3. Forest plot of early mortality rates (≤30 days) across studies [7–14,16,18–22,24,25,29,31,32,34–54,56]. This figure displays the early post-operative mortality rate from each study and the overall pooled estimate.

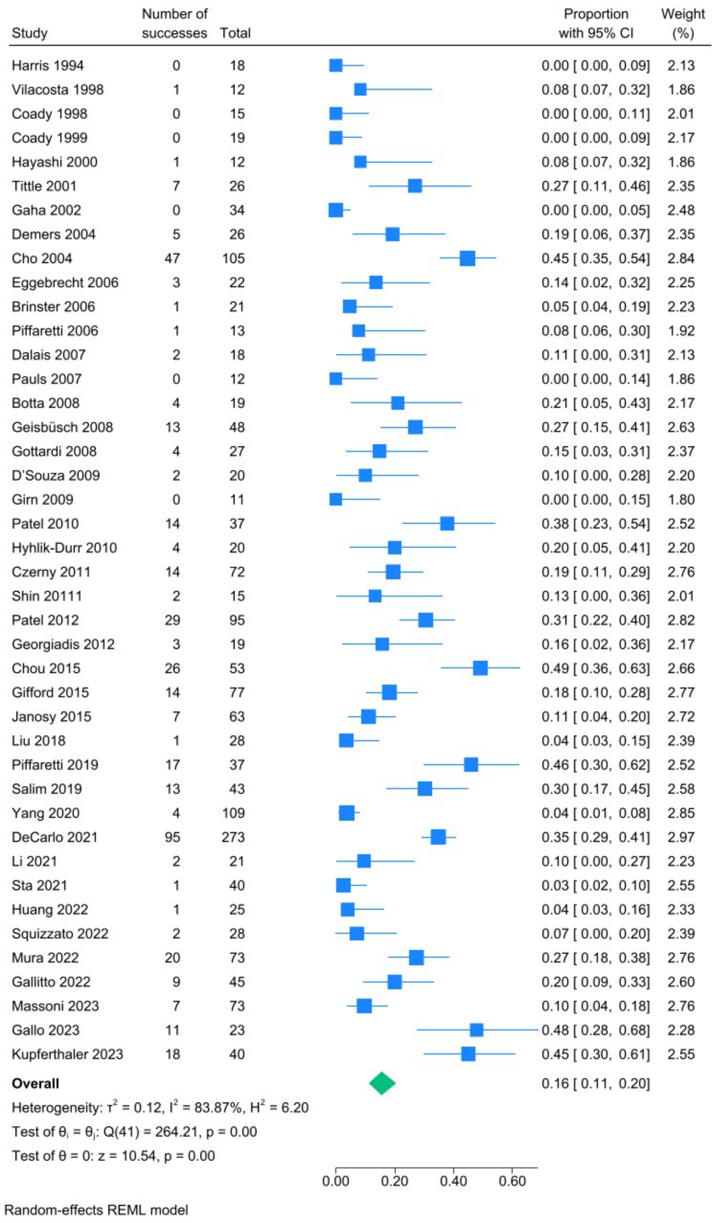

Figure S4. Forest plot of late mortality rates (>30 days) across included studies [7–14,16–18,20–22,24–26,30–32,34–36,38–56].

1. Taguchi E, Nishigami K, Miyamoto S, Sakamoto T, Nakao K. Impact of shear stress and atherosclerosis on entrance-tear formation in patients with acute aortic syndromes. Heart Vessels. Springer-Verlag Tokyo; 2014;29(1):78–82. DOI: 10.1007/S00380-013-0328-Z
2. Wada H, Sakata N, Tashiro T. Clinicopathological study on penetrating atherosclerotic ulcers and aortic dissection: distinct pattern of development of initial event. Heart Vessels. 2016;31(11):1855–61. DOI: 10.1007/s00380-016-0813-2
3. Amin MF, Abd El Gawad EA. Diagnostic performance of MDCT angiography in penetrating atherosclerotic aortic ulcer and its complications compared with digital subtraction angiography. Egyptian Journal of Radiology and Nuclear Medicine. 2016;47(3):909–17. DOI: 10.1016/j.ejrnm.2016.06.002
4. Flohr TR, Hagspiel KD, Jain A, Tracci MC, Kern JA, Kron IL, et al. The natural history of penetrating ulcers of the iliac arteries. J Vasc Surg. United States; 2016;63(2):399–406. DOI: 10.1016/j.jvs.2015.08.097

5. Flohr TR, Hagspiel KD, Jain A, Tracci MC, Kern JA, Kron IL, et al. The History of Incidentally Discovered Penetrating Aortic Ulcers of the Abdominal Aorta. Ann Vasc Surg. Netherlands; 2016;31:8–17. DOI: 10.1016/j.avsg.2015.08.028
6. Bellomo TR, DeCarlo C, Khoury MK, Lella SK, Png CYM, Kim Y, et al. Outcomes of symptomatic penetrating aortic ulcer and intramural hematoma in the endovascular era. J Vasc Surg. 2023;78(5):1180–7. DOI: 10.1016/j.jvs.2023.06.107
7. Huang B, Jia H, Lai H, Chen Z, Sun Y, Wang C. Outcomes of thoracic endovascular aortic repair for penetrating aortic ulcers involving the left subclavian artery with the Castor single-branched stent graft. European Journal of Cardio-thoracic Surgery. 2022;62(2). DOI: 10.1093/ejcts/ezac102
8. Yang L, Zhang QY, Wang XZ, Zhao X, Liu XZ, Wang P, et al. Long-Term Imaging Evolution and Clinical Prognosis Among Patients With Acute Penetrating Aortic Ulcers: A Retrospective Observational Study. J Am Heart Assoc. England; 2020;9(18):e014505. DOI: 10.1161/JAHA.119.014505
9. Eggebrecht H, Herold U, Schmermund A, Lind AY, Kuhnt O, Martini S, et al. Endovascular stent-graft treatment of penetrating aortic ulcer: results over a median follow-up of 27 months. Am Heart J. United States; 2006;151(2):530–6. DOI: 10.1016/j.ahj.2005.05.020
10. Brinster DR, 3rd WGH, Williams J, Ramaiah VG, Diethrich EB, Rodriguez-Lopez JA. Are penetrating aortic ulcers best treated using an endovascular approach? Ann Thorac Surg. Netherlands; 2006;82(5):1688–91. DOI: 10.1016/j.athoracsur.2006.05.043
11. Geisbüsch P, Kotelis D, Weber TF, Hyhlik-Dürr A, Kauczor H-U, Böckler D. Early and midterm results after endovascular stent graft repair of penetrating aortic ulcers. J Vasc Surg. 2008;48(6):1361–8. DOI: 10.1016/j.jvs.2008.07.058
12. Patel HJ, Williams DM, Upchurch Jr. GR, Dasika NL, Deeb GM. The challenge of associated intramural hematoma with endovascular repair for penetrating ulcers of the descending thoracic aorta. J Vasc Surg. 2010;51(4):829–35. DOI: 10.1016/j.jvs.2009.11.050
13. Czerny M, Funovics M, Sodeck G, Dumfarth J, Schoder M, Juraszek A, et al. Results after thoracic endovascular aortic repair in penetrating atherosclerotic ulcers. Annals of Thoracic Surgery. 2011;92(2):562–7. DOI: 10.1016/j.athoracsur.2011.02.087
14. Shin JH, Angle JF, Park AW, Anderson C, Sabri SS, Turba UC, et al. CT imaging findings and their relevance to the clinical outcomes after stent graft repair of penetrating aortic ulcers: Six-year, single-center experience. Cardiovasc Intervent Radiol. 2012;35(6):1301–7. DOI: 10.1007/s00270-011-0301-0
15. Romanowski Ł, Grochowiecki T, Szmidt J. Evaluation of the results of penetrating atherosclerotic ulcers surgical treatment. Acta Angiologica. 2013;19(1):9–17.
16. Ganaha F, Miller DC, Sugimoto K, Do YS, Minamiguchi H, Saito H, et al. Prognosis of aortic intramural hematoma with and without penetrating atherosclerotic ulcer: A clinical and radiological analysis. Circulation. 2002;106(3):342–8. DOI: 10.1161/01.CIR.0000022164.26075.5A
17. Kupferthaler A, Hauck SR, Schwarz M, Kern M, Deinsberger J, Dachs T-M, et al. Endovascular Repair of Penetrating Thoracic Aortic Ulcers Using Tubular Stent Grafts Versus Stent Grafts With a Proximal Scallop. Journal of Endovascular Therapy. 2023; DOI: 10.1177/15266028221149919
18. Gallo M, van den Berg JC, Torre T, Riggi M, Demertzis S, Ferrari E. Long-Term Outcomes and Risk Factors Analysis for Patients Undergoing Thoracic Endovascular Aorta Repair (TEVAR), According to the Aortic Pathologies. Ann Vasc Surg. 2023;94:362–8. DOI: 10.1016/j.avsg.2023.02.012
19. Hatzl J, Behrendt CA, Schmitz-Rixen T, Grundmann RT, Steinbauer M, Böckler D, et al. Outcomes of endovascular repair of infrarenal penetrating aortic ulcers: Insights from the abdominal aortic aneurysm registry of the

German Institute for Vascular Research. Vasa - European Journal of Vascular Medicine. Hogrefe Verlag GmbH & Co. KG; 2023;52(3):169–74. DOI: 10.1024/0301-1526/A001060

20. Bianchini Massoni C, Ancetti S, Perini P, Spath P, D'Ospina RM, Gallitto E, et al. Infrarenal EVAR for Penetrating Aortic Ulcer: A Comparative Study with Abdominal Aortic Aneurysm. Ann Vasc Surg. Elsevier Inc.; 2023;88:327–36. DOI: 10.1016/J.AVSG.2022.06.101
21. Gallitto E, Faggioli G, Spath P, Ancetti S, Pini R, Logiacco A, et al. Midterm results of complicated penetrating abdominal aortic ulcer treated by aortobi-iliac endograft and embolization. J Vasc Surg. Elsevier Inc.; 2023;77(1):106-113.e2. DOI: 10.1016/J.JVS.2022.07.181
22. Murana G, Di Marco L, Fiorentino M, Buia F, Brillanti G, Lovato L, et al. Endovascular treatment of penetrating atherosclerotic ulcers of the arch and thoracic aorta: In-hospital and 5-year outcomes. JTCVS Open. Elsevier B.V.; 2022;10:12–21. DOI: 10.1016/J.XJON.2022.03.003
23. Zhang R, Sun L, Sun W, Yang S, Hao Y. Endovascular Repair of Penetrating Aortic Ulcers: Indications and Single-Center Mid-Term Results. Ann Vasc Surg. 2022;81:129–37. DOI: 10.1016/j.avsg.2021.09.037
24. Stana J, C FP, Kruszyna L, Passaloglou IT, Ruffino MA, A RV, et al. Multicenter Experience With Large Diameter Balloon-Expandable Stent-Grafts for the Treatment of Infrarenal Penetrating Aortic Ulcers. J Endovasc Ther. United States; 2021;28(6):871–7. DOI: 10.1177/15266028211028221
25. Li G, Xu X, Li J, Xiong S. Thoracic Endovascular Aortic Repair for Retrograde Type A Aortic Intramural Hematoma. Front Cardiovasc Med. Frontiers Media SA; 2021;8. DOI: 10.3389/FCVM.2021.712524
26. Decarlo C, Latz CA, Boitano LT, Kim Y, Tanious A, Schwartz SI, et al. Prognostication of Asymptomatic Penetrating Aortic Ulcers: A Modern Approach. Circulation. 2021;144(14):1091–101. DOI: 10.1161/CIRCULATIONAHA.121.054710
27. Jiang X, Pan T, Zou L, Chen B, Jiang J, Shi Y, et al. Outcomes of endovascular stent graft repair for penetrating aortic ulcers with or without intramural hematoma. J Vasc Surg. United States; 2021;73(5):1541–8. DOI: 10.1016/j.jvs.2020.10.022
28. Illuminati G, Pasqua R, Nardi P, Fratini C, Calio FG, Ricco JB. Intravascular Ultrasound-Assisted Endovascular Exclusion of Penetrating Aortic Ulcers. Ann Vasc Surg. Elsevier Inc.; 2021;70:467–73. DOI: 10.1016/J.AVSG.2020.06.059
29. Kruszyna Ł, Dzieciuchowicz Ł, Strauss E, Oszkinis G. Midterm Results of the Treatment of Penetrating Abdominal Aortic or Iliac Artery Ulcer with the BeGraft Balloon-Expandable Covered Stent-A Single-Center Experience. Ann Vasc Surg. Department of General and Vascular Surgery, University of Medical Sciences, Poznan, Poland. Electronic address: lukaszkruszyna@poczta.onet.pl.; 2020;69:382–90. DOI: 10.1016/j.avsg.2020.05.056
30. Salim S, Locci R, Martin G, Gibbs R, Jenkins M, Hamady M, et al. Short- and long-term outcomes in isolated penetrating aortic ulcer disease. J Vasc Surg. United States; 2020;72(1):84–91. DOI: 10.1016/j.jvs.2019.09.039
31. Piffaretti G, Fontana F, Tadiello M, Guttadauro C, Piacentino F, Bush RL, et al. Arch and access vessel complications in penetrating aortic ulcer managed with thoracic endovascular aortic repair. Ann Cardiothorac Surg. China; 2019;8(4):471–82. DOI: 10.21037/acs.2019.06.07
32. Liu J, Liu Y, Yang W, Gu J, Xue S. Five-year outcomes after thoracic endovascular aortic repair of symptomatic type B penetrating aortic ulcer with intramural hematoma in Chinese patients. J Thorac Dis. China; 2019;11(1):206–13. DOI: 10.21037/jtd.2018.12.86
33. Gabel JA, Tomihama RT, Abou-Zamzam AM, Nekrasov V, Oyoyo UE, Bianchi C, et al. Early Surgical Referral for Penetrating Aortic Ulcer Leads to Improved Outcome and Overall Survival. Ann Vasc Surg. 2019;57:29–34. DOI: 10.1016/j.avsg.2018.12.062

34. Jánosi RA, Gorla R, Tsagakis K, Kahlert P, Horacek M, Bruckschen F, et al. Thoracic Endovascular Repair of Complicated Penetrating Aortic Ulcer. Journal of Endovascular Therapy. SAGE PublicationsSage CA: Los Angeles, CA; 2016;23(1):150–9. DOI: 10.1177/1526602815613790
35. Gifford SM, Duncan AA, Greiten LE, Gloviczki P, Oderich GS, Kalra M, et al. The natural history and outcomes for thoracic and abdominal penetrating aortic ulcers. J Vasc Surg. United States; 2016;63(5):1182–8. DOI: 10.1016/j.jvs.2015.11.050
36. Chou AS, Ziganshin BA, Charilaou P, Tranquilli M, Rizzo JA, Elefteriades JA. Long-term behavior of aortic intramural hematomas and penetrating ulcers Read at the 95th Annual Meeting of the American Association for Thoracic Surgery, Seattle, Washington, April 25-29, 2015. Journal of Thoracic and Cardiovascular Surgery. Mosby Inc.; 2016;151(2):361-373.e1. DOI: 10.1016/j.jtcvs.2015.09.012
37. Taher F, Assadian A, Strassegger J, Duschek N, Koulas S, Senekowitsch C, et al. Pararenal Aortic Ulcer Repair. European Journal of Vascular and Endovascular Surgery. W.B. Saunders Ltd; 2016;51(4):504–10. DOI: 10.1016/j.ejvs.2015.12.005
38. Georgiadis GS, Trellopoulos G, Antoniou GA, Georgakarakos EI, Nikolopoulos ES, Pelekas D, et al. Endovascular therapy for penetrating ulcers of the infrarenal aorta. ANZ J Surg. John Wiley & Sons, Ltd; 2013;83(10):758–63. DOI: 10.1111/ANS.12074;REQUESTEDJOURNAL:JOURNAL:14452197;WGROUP:STRING:PUBLICATION
39. Girn HRS, McPherson S, Nicholson T, Mavor AID, Homer-Vanniasinkam S, Gough MJ. Short series of emergency stent-graft repair of symptomatic penetrating thoracic aortic ulcers (PTAU). Vasc Med. Vasc Med; 2009;14(2):123–8. DOI: 10.1177/1358863X08098951
40. Hyhlik-Dürr A, Geisbüsch P, Kotelis D, Böckler D. Endovascular repair of infrarenal penetrating aortic ulcers: a single-center experience in 20 patients. J Endovasc Ther. United States; 2010;17(4):510–4. DOI: 10.1583/10-3063.1
41. D'Souza S, Duncan A, Aguila F, Oderich G, Ricotta J, Kalra M, et al. TEVAR for non-aneurysmal thoracic aortic pathology. Catheterization and Cardiovascular Interventions. 2009;74(5):783–6. DOI: 10.1002/ccd.22123
42. Gottardi R, Zimpfer D, Funovics M, Schoder M, Lammer J, Wolner E, et al. Mid-term results after endovascular stent-graft placement due to penetrating atherosclerotic ulcers of the thoracic aorta. European Journal of Cardio-thoracic Surgery. 2008;33(6):1019–24. DOI: 10.1016/j.ejcts.2007.12.054
43. Botta L, Buttazzi K, Russo V, Parlapiano M, Gostoli V, Di Bartolomeo R, et al. Endovascular Repair for Penetrating Atherosclerotic Ulcers of the Descending Thoracic Aorta: Early and Mid-Term Results. Annals of Thoracic Surgery. 2008;85(3):987–92. DOI: 10.1016/j.athoracsur.2007.10.079
44. Pauls S, Orend KH, Sunder-Plassmann L, Kick J, Schelzig H. Endovascular Repair of Symptomatic Penetrating Atherosclerotic Ulcer of the Thoracic Aorta. European Journal of Vascular and Endovascular Surgery. 2007;34(1):66–73. DOI: 10.1016/J.EJVS.2006.12.029
45. Dalainas I, Nano G, Medda M, Bianchi P, Casana R, Ramponi F, et al. Endovascular Treatment of Penetrating Aortic Ulcers: Mid-term Results. European Journal of Vascular and Endovascular Surgery. 2007;34(1):74–8. DOI: 10.1016/j.ejvs.2007.02.025
46. Piffaretti G, Tozzi M, Lomazzi C, Rivolta N, Caronno R, Castelli P. Endovascular repair of abdominal infrarenal penetrating aortic ulcers: A prospective observational study. International Journal of Surgery. 2007;5(3):172–5. DOI: 10.1016/J.IJSU.2006.06.004
47. Cho KR, Stanson AW, Potter DD, Cherry KJ, Schaff H V., Sundt TM, et al. Penetrating atherosclerotic ulcer of the descending thoracic aorta and arch. Journal of Thoracic and Cardiovascular Surgery. Mosby Inc.; 2004;127(5):1393–401. DOI: 10.1016/J.JTCVS.2003.11.050

48. Demers P, Miller DC, Mitchell RS, Kee ST, Chagonjian L, Dake MD. Stent-graft repair of penetrating atherosclerotic ulcers in the descending thoracic aorta: Mid-term results. Annals of Thoracic Surgery. 2004;77(1):81–6. DOI: 10.1016/S0003-4975(03)00816-6
49. Tittle SL, Lynch RJ, Cole PE, Singh HS, Rizzo JA, Kopf GS, et al. Midterm follow-up of penetrating ulcer and intramural hematoma of the aorta. J Thorac Cardiovasc Surg. United States; 2002;123(6):1051–9. DOI: 10.1067/mtc.2002.121681
50. Hayashi H, Matsuoka Y, Sakamoto I, Sueyoshi E, Okimoto T, Hayashi K, et al. Penetrating atherosclerotic ulcer of the aorta: imaging features and disease concept. Radiographics [Internet]. 2000;20(4):995–1005. Available from: https://www.embase.com/search/results?subaction=viewrecord&id=L31344443&from=export
51. Harris JA, Bis KG, Glover JL, Bendick PJ, Shetty A, Brown OW. Penetrating atherosclerotic ulcers of the aorta. J Vasc Surg. 1994;19(1):90–9. DOI: 10.1016/S0741-5214(94)70124-5
52. Coady MA, Rizzo JA, Elefteriades JA. Pathologic variants of thoracic aortic dissections: Penetrating atherosclerotic ulcers and intramural hematomas. Cardiol Clin. W.B. Saunders; 1999;17(4):637–57. DOI: 10.1016/S0733-8651(05)70106-5
53. Coady MA, Rizzo JA, Hammond GL, Pierce JG, Kopf GS, Elefteriades JA, et al. Penetrating ulcer of the thoracic aorta: What is it? How do we recognize it? How do we manage it? J Vasc Surg. Mosby Inc.; 1998;27(6):1006–16. DOI: 10.1016/S0741-5214(98)70003-5
54. Vilacosta I, San Román JA, Aragoncillo P, Ferreirós J, Mendez R, Graupner C, et al. Penetrating atherosclerotic aortic ulcer: Documentation by transesophageal echocardiography. J Am Coll Cardiol. 1998;32(1):83–9. DOI: 10.1016/S0735-1097(98)00194-6
55. Squizzato F, Hyun MC, Sen I, D'Oria M, Bower T, Oderich G, et al. Predictors of Long-Term Aortic Growth and Disease Progression in Patients with Aortic Dissection, Intramural Hematoma, and Penetrating Aortic Ulcer. Ann Vasc Surg. Ann Vasc Surg; 2022;81:22–35. DOI: 10.1016/J.AVSG.2021.10.047
56. Patel HJ, Sood V, Williams DM, Dasika NL, Diener AC, Deeb GM. Late outcomes with repair of penetrating thoracic aortic ulcers: the merits of an endovascular approach. Ann Thorac Surg. Netherlands; 2012;94(2):516–22; discussion 522-3. DOI: 10.1016/j.athoracsur.2012.03.074
